# Functionality of MC88- and MPC85-Enriched Skim Milk: Impact of Shear Conditions in Rotor/Stator Systems and High-Pressure Homogenizers on Powder Solubility and Rennet Gelation Behavior

**DOI:** 10.3390/foods10061361

**Published:** 2021-06-11

**Authors:** Malou Warncke, Ulrich Kulozik

**Affiliations:** Chair of Food and Bioprocess Engineering, TUM School of Life Sciences, Technical University of Munich, Weihenstephaner Berg 1, 85354 Freising, Germany; ulrich.kulozik@tum.de

**Keywords:** cheese manufacture, milk protein powders, rehydration, dissolution, particle size, shear rate, powder aggregates, protein accessibility, upscaling

## Abstract

Milk protein concentrate (MPC) and micellar casein (MC) powders are commonly used to increase the protein concentration of cheese milk. However, highly-concentrated milk protein powders are challenging in terms of solubility. The research question was whether and how incompletely dissolved agglomerates affect the protein functionality in terms of rennet gelation behavior. For the experiments, skim milk was enriched with either MC88 or MPC85 to a casein concentration of 4.5% (*w*/*w*) and sheared on a laboratory and pilot scale in rotor/stator systems (colloid mill and shear pump, respectively) and high-pressure homogenizers. The assessment criteria were on the one hand particle sizes as a function of shear rate, and on the other hand, the rennet gelation properties meaning gelling time, gel strength, structure loss upon deformation, and serum loss. Furthermore, the casein, whey protein, and casein macropeptide (CMP) recovery in the sweet whey was determined to evaluate the shear-, and hence, the particle size-dependent protein accessibility. We showed that insufficient powder rehydration prolongs the rennet gelation time, leading to softer, weaker gels, and to lower amounts of CMP and whey protein in the sweet whey.

## 1. Introduction

In cheese manufacture, it is common to increase the casein or the total protein concentration of the vat milk to increase the cheese yield [1,2]. There are two possibilities to vary the total protein concentration or the casein/whey protein ratio: by micro- or ultrafiltration or by adding high protein powders like micellar casein (MC) or milk protein concentrate (MPC) powders [3]. With powders the protein content can be standardized flexibly, and seasonal fluctuations of the milk composition can be compensated [3]. These are known to have a major impact on the curd forming properties and, on the composition and the yield of the final cheese [4,5,6,7]. However, the redispersion of high protein powders can be challenging in terms of solubility due to their insufficient dissolving ability [8], which is often neglected in studies assuming that rehydration overnight would ensure full powder dissolution.

According to Oldfield and Singh [9], the extent of protein interactions that occur upon pre-heating before evaporation and spray drying in milk powder production affects the powder solubility and shelf life. Crowley et al. [10] attributed the poor solubility of MPC to the mineral depletion during diafiltration. For high protein powder production, milk protein concentrates are usually diafiltered with water to wash out lactose and salts and to increase the protein concentration. Consequently, the powder shows a higher Ca^2+^-activity due to changes in the milk salt equilibrium between the aqueous and dispersed phase, which renders the powder less soluble [11,12]. In a previous study we could already show that even poorly dissolvable MC and MPC85 were fully solubilized with high-pressure homogenization (HPH) resulting in the particle size distribution (PSD) of fresh skim milk [13]. Furthermore, we found out that remaining powder particles increase the shear stress of reconstituted milk protein concentrates. However, it was not investigated so far whether and how incompletely dissolved powder agglomerates affect the protein functionality in terms of rennet gelation behavior.

Ferrer et al. [14] investigated MPC56, MPC70, and MPC90 regarding their renneting properties. The authors reported that the gel strength of MPC90 was lower after 3 h oscillating in the linear viscoelastic region (LVR) compared to MPC56 and MPC70. This is not surprising considering the particle sizes of the powders, which were 0.14 µm in MPC56 and MPC70 samples compared to 0.92 µm in the MPC90 sample. Martin et al. [15] observed an increased strength of rennet gels made of reconstituted milk powders in water with increasing reconstitution time. Upon adding at least 1 mM calcium chloride the gel strength massively increased with increasing calcium chloride concentration. This led the authors to the assumption that some calcium in the environment (added in pure form or released from the powder particles) is necessary to induce aggregation. They suspected that the incubation time might have been too short to achieve full calcium release from the powder particles resulting in softer gels. This can also be explained with insufficient powder rehydration; it can be assumed that calcium may dissolve faster the better the powder particles were rehydrated. All three studies performed the powder dissolution on a laboratory scale. We wanted to investigate whether the shear impact on a laboratory scale is comparable to that on a pilot scale when applying the same shear rates. However, the transferability to the industrial scale remains to be demonstrated.

Many different low and high shear systems can be found in literature for powder redispersion at the laboratory scale: magnetic stir bars [10,16,17,18], overhead stirrers equipped with different stirrer blade geometries [1,10,19,20,21], handheld homogenizers [14,22], ultrasonication [22], as well as HPH [13,22]. Chandrapala et al. [22] examined the effect of different shear systems on the solubility of MPC80 and MC. They compared the efficiency of low shear (overhead stirrer), high shear (handheld homogenizer), ultrasonication, and HPH not at the same shear rate but at the same input energy density. The authors showed that the handheld homogenizer did not significantly downsize the PSD of MPC90 and MC. Only HPH shifted the PSD towards casein micelle size, although, the particle sizes were still bimodally-distributed. However, the lowest necessary shear impact for full MPC90 and MC dissolution has not been identified. Since a handheld homogenizer does not run continuously, it is hardly transferable to industrial shear systems.

In the dairy industry, two high shear units are widely implemented and can be used for powder redispersion: shear pumps like in powder mixers and HPH. The shear conditions, such as shear rate, turbulent flow, and cavitation (only in HPH) are involved in particle destruction. Therefore, we performed experiments on a laboratory scale first, using a colloid mill as rotor/stator system and a laboratory scale HPH. For the subsequent upscaling experiments a shear pump and a pilot scale HPH were used.

To the best of our knowledge, a study dealing with the impact of high shear conditions on the protein functionality MC- and MPC-enriched skim milk in terms of rennet gelation behavior has never been reported so far. We hypothesized that remaining powder particles of poorly soluble MC88 and MPC85 impair the rennet gelation behavior due to inaccessible proteins in the powder aggregates as well as the gel properties, which could be disturbed by large powder particles.

To evaluate the impact of remaining powder aggregates on the protein functionality, the rennet gelation behavior of the MC88- and MPC85-enriched skim milk was investigated. MC88 and MPC85 differ in protein composition and presence of low molecular solutes, which are known to have an impact on speed and completeness of milk powder rehydration. For the experiments, skim milk was enriched with either MC88 or MPC85 to a casein concentration of 4.5% (*w*/*w*), which is in the range typical for cheesemaking [23]. The assessment criteria were particle size as a function of shear rate and the rennet gelation properties. As for cheese manufacture, gelling time, gel strength, structure loss upon deformation, and serum loss were measured. Furthermore, the casein, whey protein, and casein macropeptide (CMP) recovery in the sweet whey was determined to evaluate the shear-, and hence, particle size-dependent protein accessibility.

This study should provide insights into the effect of incomplete rehydration of added milk protein powders of various composition on the rennet gelation behavior of powder-enriched skim milk. This enables producing cheese from protein-enriched milk without filtration, but with the same characteristics as retentates. Depending on the powder type, the composition can be varied, and the functionality of the protein-enriched milk can be enhanced. With upscaling experiments, we can recommend suitable shear conditions for best powder dissolution and protein functionality on laboratory as well as on pilot scale.

## 2. Materials and Methods

### 2.1. MC88- and MPC85-Enriched Skim Milk

Pasteurized skim milk (74 °C, 28 s) from the local dairy (Molkerei Weihenstephan GmbH & Co. KG, Freising, Germany) was either enriched with micellar casein concentrate powder containing 88% (*w*/*w*) total protein (MC88) or with milk protein concentrate powder containing 85% total protein (MPC85) purchased from MILEI GmbH, Leutkirch im Allgäu (commercial name TMP85). Both powders were used before the best before date. The casein concentration was adjusted to 4.5% (*w*/*w*), which is in the range typical for cheesemaking [23]. The powder compositions are presented in Table 1.

Casein and whey protein content (according to Dumpler et al. [24]) and lactose content (according to Schmitz-Schug et al. [25]) were determined by reversed-phase high performance liquid chromatography (RP-HPLC). The protein concentrations given in Table 1 represent the pure protein fractions without peptides; hence, the total protein content, which consists of both, protein fractions and peptides, is lower than 88 and 85%. The flame photometer ELEX 6361 (Eppendorf AG, Hamburg, Germany) was used to measure the amount of soluble minerals (Na^+^, K^+^) and the total Ca^2+^ concentration in the rehydrated powders. All powders were stored in aluminum compound foil bags at 20 °C to avoid oxygen migration through the packaging material and to prevent the powder from UV radiation.

### 2.2. Shear Treatments

The impact of shear in a rotor/stator system as well as in a high-pressure homogenizer (HPH) on the particle size was investigated. For shear treatments on a laboratory scale a colloid mill (IKA Laboratory Pilot 2000/4, IKA-Werke, Staufen im Breisgau, Germany) equipped with a radial impeller MK module and a laboratory HPH (APV 1000, SPX Flow Technology, Crawley West Sussex, UK) were used. The corresponding systems in the upscaling experiments were the shear pump FSP 712/124 (FRISTAM Pumpen KG (GmbH & Co., Hamburg, Germany) and the pilot HPH Rannie 56 type 16.56H with a single stage valve (APV Gaulin GmbH, Lübeck, Germany). In all cases, the samples were sheared in single path, imitating an industrial, continuous process.

Before the shear treatments, one batch each of MC88- and MPC85-enriched skim milk, respectively, was produced. For this, the powders were pre-dissolved in the milk for 30 min at 40 °C under steady stirring at 27 s^−1^. At pilot scale level, we produced 200 kg pre-mixture in a tempered double-walled cream maturing tank (*d_T_* = 80 cm, Inox Behälter GmbH, Delmenhorst, Germany) equipped with an anchor stirrer (*d_i_* = 76 cm, *W* = 6.4 cm). On laboratory scale, we produced 1300 g using a metal tank (*d_T_* = 16 cm) placed in a water bath and stirred with an overhead stirrer (IKA MINISTAR 80 digital, IKA-Werke, Staufen im Breisgau, Germany) equipped with an anchor stirrer as well (*d_i_* = 15 cm, *W* = 2 cm). The shear rate $\dot{\gamma }$ (s^−1^) in a stirred tank was calculated by Equation (1) according to Bowen [26]:

(1) $$\dot{\gamma }=4.2\cdot N\cdot {\left(\frac{d_{i}}{d_{T}}\right)}^{0.3}\cdot \frac{d_{i}}{W}$$

where N was the stirrer speed (s^−1^), $d_{i}$ was the diameter of the stirrer (m), $d_{T}$ the diameter of the tank (m), W the width of the stirrer blade (m), and 4.2 and 0.3 were empirically determined factors.

The samples were treated at four different shear rates in the colloid mill/shear pump which was determined according to Equations (2) and (3).

(2) $$\dot{\gamma }=\frac{dv}{dh}=\frac{2v}{h}$$

where v is the flow velocity (m s^−1^) and h the gap width (m). v is defined as

(3) v=2πrf

where r is the radius of the stator (m) and f the frequency of the rotor (s^−1^).

The shear rate, which induces the aggregates’ destruction in the high-pressure homogenizer gap can be calculated by Equation (4):

(4) $$\dot{\gamma }=\frac{2\;\dot{V}}{\pi \;d_{eff\;}h^{2}}$$

$\dot{V}$ is the flow rate (m^3^ s^−1^), $d_{eff}$ the efficient diameter (m) and h the gap height (m).

The configurations for the desired shear rate of the stirred tanks, colloid mill, shear pump, and high-pressure homogenizers are presented in Table 2.

**Figure 1:**
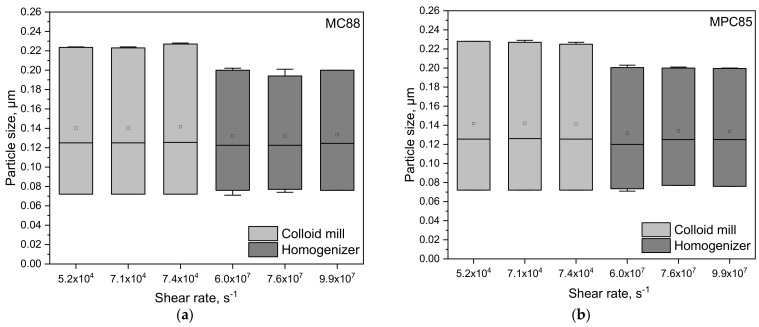
d_10_, d_50_, and d_90_ represented as boxplots of skim milk enriched with MC88 (**a**) and MPC85 (**b**) after homogenization with colloid mill and high-pressure homogenizer at shear rates ≥ 5.2 × 10^4^ and ≥6.0 × 10^7^ s^−1^, respectively. The low and high quartiles, the median, and the mean (□) are plotted.

The lowest possible rotation speed for the colloid mill was 3170 min^−1^; hence, the minimum possible shear rate was 3.3 × 10^3^ s^−1^. In preliminary experiments, it turned out that after treating the samples at shear rates higher than 5.2 × 10^4^ s^−1^ and 6.0 × 10^7^ s^−1^, respectively, the particle sizes did not change with increasing shear rate (Figure 1). Therefore, the maximum shear rate chosen for the rotor/stator system was 5.2 × 10^4^ s^−1^ and for the high-pressure homogenizer, it was 6.0 × 10^7^ s^−1^ (corresponding to 100 bar). Each experiment was performed in duplicate.

### 2.3. Particle Size Measurements

Particle sizes were measured by static light scattering using a Malvern Mastersizer 2000 equipped with a Malvern Hydro 2000S sample dispersion unit (Malvern Instruments GmbH, Herrenberg, Germany). Particle sizes are calculated within 0.02–2000 µm of up to 100 size classes applying Mie theory [27,28]. This allows measuring on the one hand casein micelles and on the other hand large powder particles at once. This method has been used by many studies for similar purposes [10,13,14,17,22,29,30,31,32]. Hence, it is established for measuring milk powder solubility.

The refractive indices of the dispersant (deionized water) and the protein were 1.33 and 1.41, respectively. The particle absorption index was 0.001 (according to Dumpler et al. [33]). The sample was added and dispersed at a constant stirrer speed (2000 rpm) until the obscuration reached 15 ± 1% according to the guidelines of the manufacturer. The stirring prevents the large powder particles from sedimentation. Besides the distribution density q_3_(x) and the cumulative distribution Q_3_(x), the software calculates the related d_10,3_, d_50,3_, and d_90,3_ values, meaning 10, 50, and 90% of the particles are smaller than the respective d-value. Fresh skim milk was the reference. Each sample was measured in duplicate at 20 °C within 3 min.

### 2.4. Rennet Gelation Properties

To evaluate the impact of remaining powder aggregates on the protein functionality, the rennet gelation behavior of the MC88- and MPC85-enriched skim milk was investigated. For this, the MCR 702 rheometer (Anton Paar GmbH, Graz, Austria) equipped with the concentric cylinder geometry CC27 was used. Of the unrenneted sample, 14.7 mL was tempered to 40 °C and simultaneously sheared at 100 s^−1^ for 5 min to give the sample time to equilibrate. After reaching and holding 40 ± 0.02 °C for 1 min, the rennet (CHY-MAX^®^ M 1000, Chr. Hansen A/S, Hørsholm, Denmark) with an enzyme activity of 1000 IMCU L^−1^ (international milk clotting units) was added with a concentration of 2.303 µL per gram casein. The rennet was mixed in immediately by shearing the sample at 500 s^−1^ for 10 s before resting for 3 s. Oscillation at a constant deformation (0.01%) and frequency (1 Hz) was applied for 30 min, whereby the sample formed a gel. The onset of gelation was defined as the point where the storage modulus G′ exceeded 1 Pa. The gelation time was defined as the time until the onset of gelation occurred.

Thirt-three minutes after rennet addition, the oscillation followed an amplitude sweep (logarithmic ramp from 0.01 to 100% deformation at a constant frequency of 1 Hz) to determine the gel strength in the linear viscoelastic region (LVR) (corresponding to 0.01–0.1% deformation within 2.5 min) and the structure loss upon deformation. The gel strength corresponded to the mean value of the measured storage modulus G′ (Pa) between 0.01–0.1% deformation. G′ is defined as the elastic portion of a sample which increases with increasing gel strength. Therefore, it can be directly related to the gel strength obtained by oscillatory measurements. The structure loss is of interest for the curd cutting, which should be smooth-running. The structure loss was calculated as follows (Equation (5)):

(5) $$Structure\;loss\;\left(\%\right)=\frac{{G^{\prime }}_{100\%}}{{G^{\prime }}_{0.01\%}}\cdot 100\%$$

where ${G^{\prime }}_{0.01\%}$ is the storage modulus at 0.01% deformation (Pa) and ${G^{\prime }}_{100\%}$ is the storage modulus at 100% deformation (Pa). All samples were measured in duplicate.

### 2.5. Serum Loss and Casein, Whey Protein, and Casein Macropeptide Recovery in the Sweet Whey

The whey drainage or serum loss, which occurs upon curd cutting and pressing, is an important criterion in cheese manufacture which defines the dry matter and therefore, the hardness of the final cheese. For serum loss determination, 30 ± 0.9 g of the unrenneted samples were weighed in duplicate into 50 mL centrifuge tubes and placed in a 40 °C-tempered water bath. After reaching 40 ± 0.1 °C, rennet was added in the same concentration as used for the rheological measurements. After 1 h of incubation, the samples were centrifuged at 4000× *g* for 45 min at 20 °C. The supernatant was immediately weighed, and the serum loss calculated by Equation (6), where $m_{serum}$ (g) is the serum mass and $m_{0}$ (g) the mass of the whole sample before renneting.

(6) $$Serum\;loss\;\left(\%\right)=\frac{m_{serum}}{m_{0}}\cdot 100\%$$

To evaluate whether the protein accessibility is impaired due to remaining nor fully rehydrated powder aggregates, the casein, whey protein, and casein macropeptide (CMP) concentrations in the sweet whey were analyzed by RP-HPLC. The protein recovery (%) indicates how much of each protein fraction found in the MC88 and MPC85-enriched skim milk was found in their supernatants after renneting and centrifuging. Since unrenneted skim milk does not contain CMP, the CMP concentrations of the renneted high-pressure-homogenized and fully solubilized MC88- and MPC85-enriched skim milks were set as relative values for CMP recovery calculation. We expected that more complete powder solubilization results in a better protein accessibility. Hence, the concentrations of whey protein and CMP in the serum phase should be higher.

### 2.6. Statistical Analyses

Origin 2020 (OriginLab Corporation, Northampton, MA, USA) was used to plot graphs and RStudio, Inc., 2019 (version 1.2.5033, Boston, MA, USA) was used for statistical analysis. Statistical significances were evaluated using one-way analysis of variance (ANOVA) combined with Tukey’s HSD post-hoc test. The calculated *P*-values and the respective significance levels are given in the text (*p* ≤ 0.001, *p* ≤ 0.01, *p* ≤ 0.05, *p* ≤ 0.1).

## 3. Results and Discussion

### 3.1. Particle Size Distributions of Protein-Enriched Skim Milk as a Function of Shear Rate

To evaluate the impact of high shear applied with colloid mill (3.3 × 10^3^–5.2 × 10^4^ s^−1^) and high-pressure homogenizer (HPH) (6.0 × 10^7^ s^−1^) on the powder solubility, we measured the particle sizes after the shear treatments by static light scattering.

**Figure 2:**
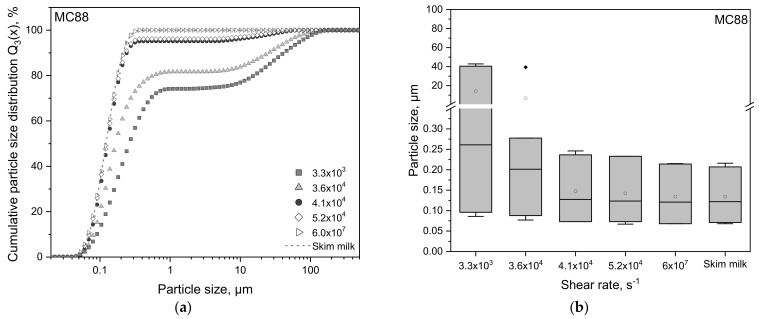

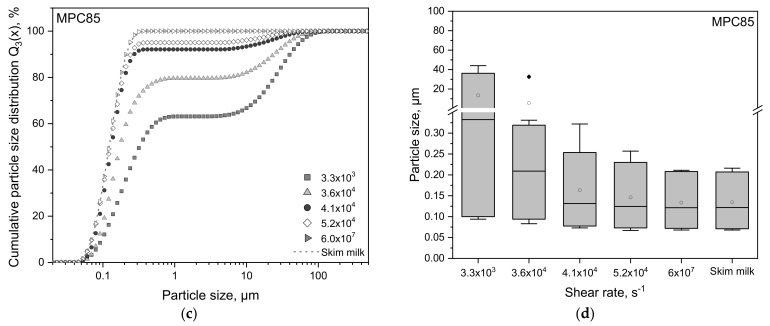
Cumulative particle size distributions Q_3_(x) (volume % vs. diameter) (left) and d_10,3_, d_50,3_, and d_90,3_ presented as boxplots (right) of skim milk and skim milk enriched with MC88 (**a**,**b**) and MPC85 (**c**,**d**) after homogenization with colloid mill (3.3 × 10^6^–5.2 × 10^4^ s^−1^) and high-pressure homogenizer (6.0 × 10^7^ s^−1^) at 40 °C. Boxplots: The low and high quartiles, the median, and the mean (□) are plotted. (♦) represents outliers.

Figure 2 presents the cumulative particle size distributions (PSD) and the d_10,3_, d_50,3_, and d_90,3_ values illustrated as boxplots for skim milk enriched with MC88 (a,b) and MPC85 (c,d) in comparison to skim milk without powder addition as a reference. The target was to achieve the same monomodal PSD as the reference, which represents the PSD of the natural casein micelles. The particle size distribution of the fresh skim milk was in accordance with Dumpler et al. [24] and Sandra and Corredig [31] (Figure 2a,c). The corresponding d_10,3_, d_50,3_, and d_90,3_ values were 70, 124, and 212 nm, respectively (Figure 2b,d). The volume-weighted mean diameter d_4,3_ was 133 nm, which was also reported by Ferrer et al. [14] and Warncke and Kulozik [13]. As expected, the particle sizes in MC88- as well as in MPC85-enriched skim milk decreased with increasing shear rate. 3.3 × 10^3^ and 3.6 × 10^4^ min^−1^ showed the worst dissolution results as their mean particle sizes were around 30 µm and their d_50,3_ varied between 0.4 and 0.2 µm (Figure 2b,d). A shear rate of at least 4.1 × 10^4^ s^−1^ markedly shifted the PSD towards smaller particle sizes. The mean particle size in both samples conformed to casein micelles (~0.15–0.2 µm). However, even the highest shear in the colloid mill (5.2 × 10^4^ s^−1^) was insufficient to fully dissolve the powders as the distributions were still bimodal (Figure 2a,c). As already shown in Section 2.2, a further shear rate increase did not improve the powder dissolution. Only HPH was able to destruct all remaining agglomerated powder particles and to achieve a monomodal particle size distribution as found for skim milk with a corresponding d_50,3_ of 0.124 µm. This leads to the assumption that laminar or turbulent flow alone, even at high shear rates, is insufficient to completely disintegrate MC88 and MPC85 powder agglomerates and that cavitation, as it occurs in HPH, is a decisive factor for solubilizing high protein powders.

MC88 was slightly better soluble than MPC85. It is said that caseins are the so-called slow dissolving components in milk protein powders, whereas whey proteins and lactose are considered as fast dissolving components. Warncke and Kulozik [13] identified lactose as the deciding fast dissolving component regarding powder solubility. It is important to note that the better soluble MC powder used in that study contained as much casein as the used MPC85, but 13.4% lactose compared to 4% in the MPC85. In the present study, the lactose concentrations of both powders were low; 1.4 and 2.7%, respectively. Although MC88 contained less fast dissolving components than MPC85, it showed a better rehydration behavior in the shear range of 3.3 × 10^3^ to 4.1 × 10^4^ s^−1^. Since the protein as well as the lactose concentrations in both powders were similar, our findings indicate that the casein/whey protein ratio, which in turn defines the extent of protein interactions and aggregate formation, determines the powders’ dissolution behavior.

Protein interactions and whey protein denaturation occurring during powder processing may be involved. According to Oldfield and Singh [9], the extent of protein interactions that occur upon preheating before evaporation and spray drying in milk powder production affects the powder solubility and shelf life. The higher the degree of whey protein denaturation and aggregation, the better the oxidative stability of whole milk powders and the worse their solubility. Oldfield et al. [34] could show that evaporating skim milk up to 49% total solids and heat treating the concentrate at 64–74 °C did not significantly affect the whey protein denaturation. The authors explained this by the increased stability of the whey proteins at high total solid contents. We postulate that this could also be related to the high lactose concentration instead of the total solid content, and therefore, to the constantly high lactose/protein ratio during evaporation. In the presence of sugars, whey proteins prefer the associated form to avoid unfavorable water–protein interactions [35,36]. Thus, lactose has a protective effect on β-lactoglobulin against denaturation if the lactose/protein ratio is high enough [37,38,39,40,41]. However, milk protein concentrates for high protein powder production are not pre-heated, but usually evaporated and spray-dried directly after diafiltration [2]. In the case of diafiltered milk protein concentrates the lactose concentration is much lower. Warncke et al. [42] observed no differences in the degree of whey protein denaturation of 0, 37, and 88% whey protein-depleted milk protein concentrates (diafiltered with ultrafiltration permeate) at the same total protein concentration after heating for 30 min at 80 °C. This was explained by the unaltered amount of reactive binding sites for the whey proteins (other whey proteins as well as the surfaces of casein micelles) in all three samples at the same total protein concentration.

As a result, the collision probability of the whey proteins with either casein micelles or other whey proteins was similar. Thus, differences in the degree of whey protein denaturation cannot be attributed to the casein/whey protein ratio alone. Warncke et al. [42] observed as well that a high whey protein ratio went hand-in-hand with an extensive whey protein aggregate growth in the serum phase. The strong disulfide bonds between the whey proteins and between the whey proteins and the κ-casein seem to be responsible for the poorer solubility of MPC85 compared to MC88 although the whey protein denaturation degree in MPC85 was 42.7 ± 0.0% and in MC88 65.4 ± 0.8%. Since this is a ratio, it is not indicating the number of formed aggregates and is therefore not a suitable measure to evaluate the powder solubility. Considering the casein/whey protein ratio as a factor could be more revealing.

The more whey proteins a powder contains, the more heat-induced aggregates form upon heating (in the evaporator and spray dryer), and the worse is the powder solubility. Mc Kenna [43] and Havea [20] identified the insoluble material in MPC85 predominantly as fused α_s_- and β-caseins, forming a skin-like structure on the powder particles’ surface, which inhibits the water penetration. Disulfide-linked κ-casein/β-lactoglobulin complexes were present as well but not considered to play an important role in the formation of insoluble material. Our results presented in Figure 2 indicate that whey protein aggregates as well as casein/whey protein complexes, which are more present in MPC85 than in MC88, are less soluble than casein aggregates, and are, hence, the least dissolving components. Therefore, MPC85 required higher shear forces for aggregates’ destruction and fully powder solubilization than MC88.

### 3.2. Functionality of MC88- and MPC85-Enriched Skim Milk: Rennet Gelation Behavior

In the following chapter, the rennet gelation behavior of MC88- and MPC85-enriched skim milk is presented.

#### 3.2.1. Gelation Time

**Figure 3:**
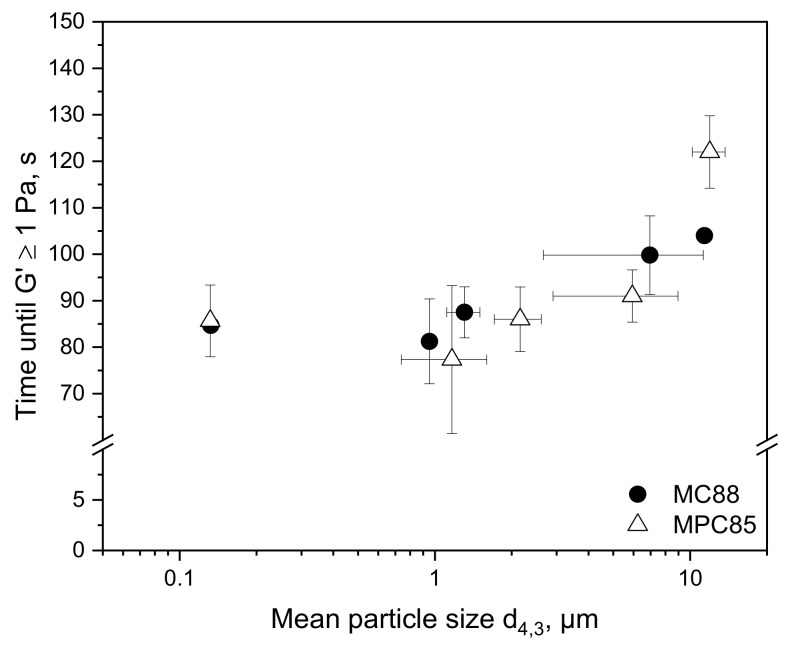
Time until the onset of gelation (G′ ≥ 1 Pa) of skim milk enriched with MC88 and MPC85 as a function of mean particle size d_4,3_.

The gelation time is defined as that time, which the gelling system requires to reach a storage modulus G′ of 1 Pa (≙ onset of gelation). Figure 3 illustrates the gelation time as a function of the mean particle size d_4,3_.

As shown before, the particle size decreased with increasing shear rate (Figure 2). Therefore, the largest particles correspond to the lowest shear rate and the smallest particles to the highest shear rate. Figure 3 shows that the gelation time decreased with decreasing particle size. MPC85 required ~120 s to reach the onset of gelation when sheared at 3.3 × 10^3^ s^−1^ (mean particle size around 10 µm) and MC88 needed ~115 s at the same shear rate and particle size. On the contrary, HPH led to a mean particle size of 0.13 µm in both powder-enriched milks and reduced the gelation time to 85 s. This is in accordance with Martin et al. [44], who observed a faster rennet gelation of reconstituted skim milks increasing in rehydration time. Although the particle sizes were not measured, it can be assumed that the particle size was also decreasing with increasing rehydration time. These results prove that insufficient powder rehydration prolongs the rennet gelation time, with the result that the curd may be cut too early in the renneting process. This may lead to fluctuations in the resulting cheese properties and quality.

The impact of whey protein denaturation on the gelation time is negligible when adding MPC85 to skim milk in that concentration used in this study since MPC85-enriched skim milk did not show significantly longer gelation times (*p* ≤ 0.1) than MC88-enriched milk.

#### 3.2.2. Gel Strength and Casein, Whey Protein, and CMP Recovery in the Sweet Whey

**Figure 4:**
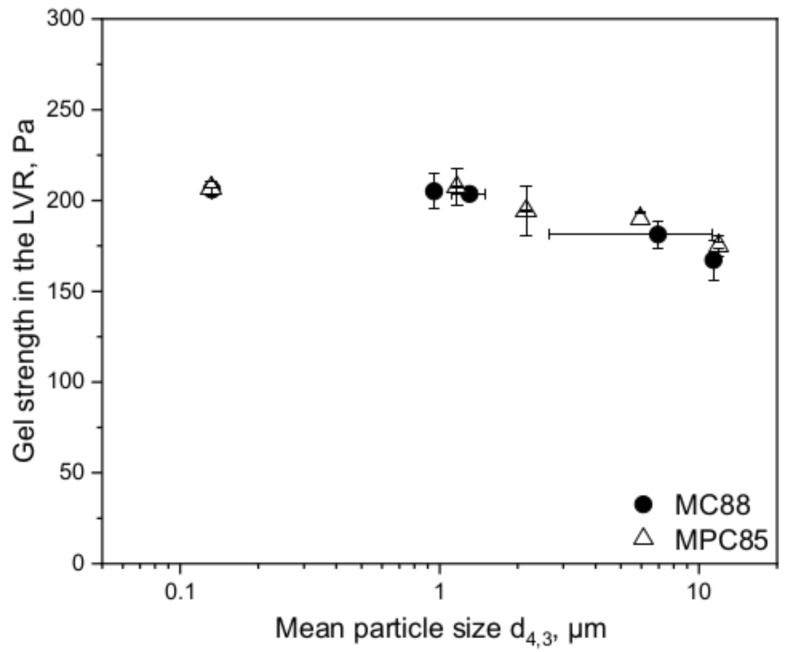
Gel strength in the linear viscoelastic region (LVR) of skim milk enriched with MC88 and MPC85 as a function of mean particle size d_4,3_. Particle sizes decrease with increasing shear rate.

The gel strength in the linear viscoelastic region (LVR) was determined by rheometry. This method provides insights regarding gel firmness without disturbing the gel network like in penetration measurements. The gel strength displays the curd firmness after full formation before cutting. We observed that the gel strength in the LVR increased with decreasing particle size and hence, increasing shear rate (Figure 4).

Our results indicate that the gel strength is particle size-dependent since we could show that the gelling time and gel strength of skim milk enriched with powders, whose whey protein denaturation degrees were around 50%, can be enhanced by higher shear treatments and particle size reduction. Hence, the insufficient rehydration of MPC90 in the study of Ferrer et al. [14] and therefore, the impaired casein micelles’ accessibility for the rennet may be responsible for the lower gel strength rather than the whey protein denaturation itself. The lower CMP release of MPC90 compared to MPC56 and MPC70 observed by Ferrer et al. [14] supports our suggestion.

We further investigated the protein concentration (casein, whey protein, and CMP) in the whey after renneting as well to evaluate the protein accessibility as a function of shear rate and particle size. On the one hand, smaller particle sizes should improve the transition of the whey proteins into the sweet whey; on the other hand, the CMP concentration should increase as well due to the separation of the casein micelles from the casein micelle agglomerates in the powder particles.

**Figure 5:**
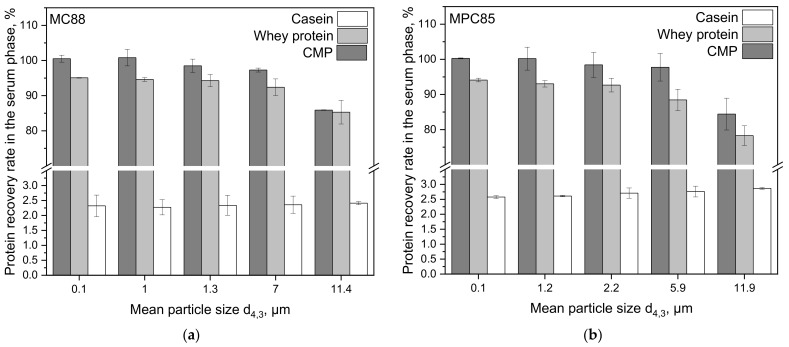
Casein, whey protein, and casein macropeptide (CMP) recovery in the serum phase after incubation (t = 1 h, ϑ = 40 °C) and centrifugation at 4000× *g* (t = 45 min, ϑ = 20 °C) of skim milk enriched with MC88 (**a**) and MPC85 (**b**) plotted for each mean particle size d_4,3_.

Figure 5 illustrates the casein, whey protein, and CMP recovery in the sweet whey as a function of particle size.

In both samples, we observed an increased whey protein and CMP recovery in the whey with decreasing particle size, which meets our expectations. Increasing the shear rate from 3.3 × 10^3^ s^−1^ to 3.6 × 10^4^ s^−1^ resulted in smaller particles but also in a significantly higher (*p* ≤ 0.01) whey protein and CMP recovery in the sweet whey. The casein recovery did not significantly change with increasing shear rate (*p* ≤ 0.1) and was constantly around 2.5% in both samples. The CMP recovery is a measure for the degree of hydrolysis. Since the CMP concentrations of the samples sheared at 6.0 × 10^7^ s^−1^ were set as relative, maximum possible values, the CMP recovery was 100% in both samples at that shear rate. It turned out that none of the samples treated at lower shear rates achieved 100% CMP recovery. This implies that a notable, particle size-dependent amount of κ-casein was not hydrolyzed, and the related CMP was consequently not found in the supernatant resulting in its lower recovery. These findings prove that the protein recovery depends on the rehydration level of the added powders only. Or, in other words, on the powder particle destruction and consequently on the better casein micelles’ accessibility for the rennet. Native whey proteins are not involved in the rennet gelation process, but most of them drain with the sweet whey. A small amount remains in the pores of the network. Their recovery in the sweet whey is also a measure for evaluating the powder solubility. The more whey proteins remain bound in powder particles, the more are involved in the gel network and the less are freely soluble in the whey and drain with the serum phase.

#### 3.2.3. Structure Loss upon Deformation and Serum Loss

After curd formation within a given set-to-cut time, the curd is cut to induce serum loss/syneresis. The serum loss defines the cheese moisture and depends on the cube size, cooking temperature, and applied pressure. It is important that the curd has the optimal firmness so it can withstand the mechanical action of the cutting knives without shattering [45]. A too soft or rigid texture causes shattering and curd fines formation (particles < 1 mm) which get lost with the whey [45]. Consequently, the cheese yield decreases.

**Figure 6:**
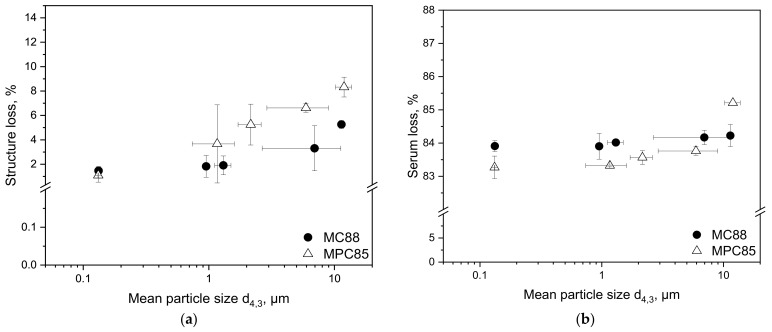
Structure loss during amplitude sweep ranging from 0.01 to 100% deformation (**a**) and serum loss after incubation (t = 1 h, ϑ = 40 °C) and centrifugation at 4000× *g* (t = 45 min, ϑ = 20 °C) (**b**) of skim milk enriched with MC88 and MPC85 as a function of mean particle size d_4,3_. Particle sizes decrease with increasing shear rate.

The structure loss is a measure for the fragility of the rennet gel. The structure loss upon deformation determined by rheometry is presented in Figure 6a.

In both samples, the structure loss decreased with increasing shear rate and decreasing particle size. If the powders are not completely dissolved (particle sizes > 0.124 µm), the MPC85-enriched skim milk show higher structure losses than the MC88 samples. The structure loss allows conclusions to be drawn about the gel strength or deformation resistance outside the LVR. The results indicate that MC88-enriched skim milk has a higher deformation resistance than MPC85-enriched skim milk. The reason is the slightly higher calcium concentration of 2.4% in MC88 compared to 2.1% (*w*/*w*) in MPC85. The dissolution medium’s composition (fresh skim milk in this case) was constant for both powders. Calcium is involved in the gel formation by forming calcium bridges between the renneted casein micelles, which in turn, defines the final curd firmness [46,47]. A low calcium concentration in the milk goes hand in hand with less calcium bridges and consequently, with a higher structure loss upon deformation. However, after HPH the structure loss of the MC88 and MPC85 samples aligned (~1.5% in both samples) at a particle size of ~0.1 µm. These results clearly demonstrate that renneted MC88- as well as MPC85-enriched skim milk become more resistant against deformation with decreasing particle size, whereas larger particle sizes result in a more fragile gel network. It could be expected that the brittle texture of the MPC85 samples homogenized with the colloid mill results in a higher serum loss because this is favored by a large surface area.

Figure 6b illustrates the serum loss of MC88- and MPC85-enriched skim milk gels after centrifugation. The serum loss of both samples decreased with decreasing particle size. As expected, MPC85-enriched skim milk showed at a d_4,3_ of ~10 µm the highest serum loss with 85.2%. Between 0.1 and 8 µm the serum loss varied between 84 and 83%. The differences between MC88 and MPC85 were insignificant (*p* ≤ 0.1).

### 3.3. Transferability from Laboratory to Pilot Scale: Correlation between Shear Rate and Particle Size

**Figure 7:**
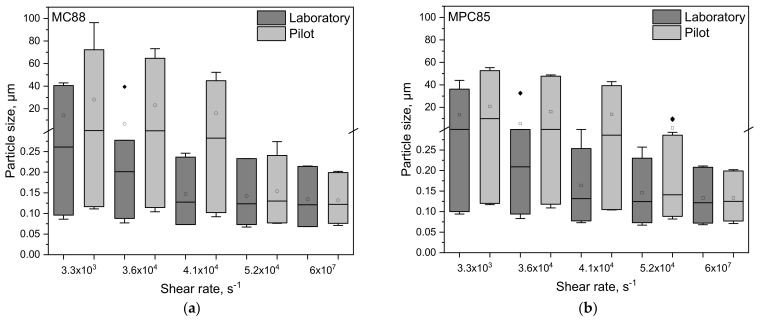
d_10,3_, d_50,3_, and d_90,3_ values observed in laboratory (dark grey) and pilot scale (light gray) presented as boxplots of skim milk enriched with MC88 (**a**) and MPC85 (**b**). The low and high quartiles, the median, and the mean (□) are plotted. (♦) represents outliers.

In order to demonstrate that the results found on a laboratory scale are transferable to pilot scale, we performed upscaling experiments. We wanted to assess whether the particle sizes obtained on a laboratory scale are obtained on a pilot scale as well when homogenizing the samples at the same shear rates. For this, a shear pump as rotor/stator system and a pilot HPH was used. However, it turned out that the particle sizes in MC88- and MPC85-enriched skim milk treated in the shear pump are higher compared to the particle sizes after colloid mill treatment in laboratory scale (Figure 7a,b). Above 4.1 × 10^4^ s^−1^ the d_10,3_, d_50,3_, and d_90,3_ values of MC88-enriched skim milk homogenized on a laboratory and a pilot scale were similar (Figure 7a). On the contrary, even the harshest conditions in the shear pump were insufficient to completely dissolve MPC85 (Figure 7b). Only HPH at 100 bar led to the same particle sizes as found on a laboratory scale.

**Figure 8:**
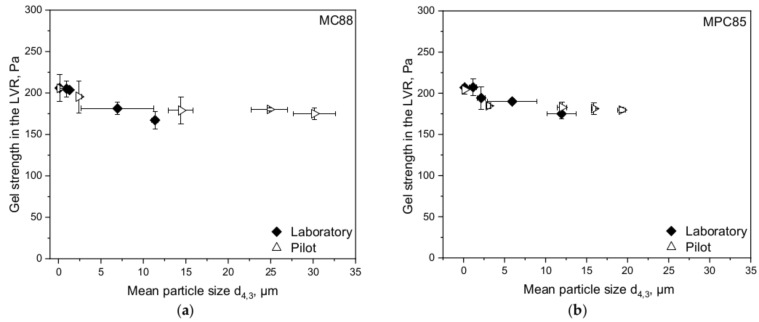
Gel strength in the linear viscoelastic region (LVR) of skim milk enriched with MC88 (**a**) and MPC85 (**b**) produced in laboratory and pilot scale as a function of mean particle size d_4,3_. Particle sizes decrease with increasing shear rate.

The larger particle sizes indicate that the powder particles undergo less shear in the pump than in the laboratory colloid mill although the shear rates were calculated in the same way. Nevertheless, the rennet gelation behavior was in accordance with the laboratory results. Figure 8 shows exemplarily for the gelling behavior the gel strength in the LVR of MC88- and MPC85-enriched skim milk sheared in laboratory and pilot scale.

**Figure 9:**
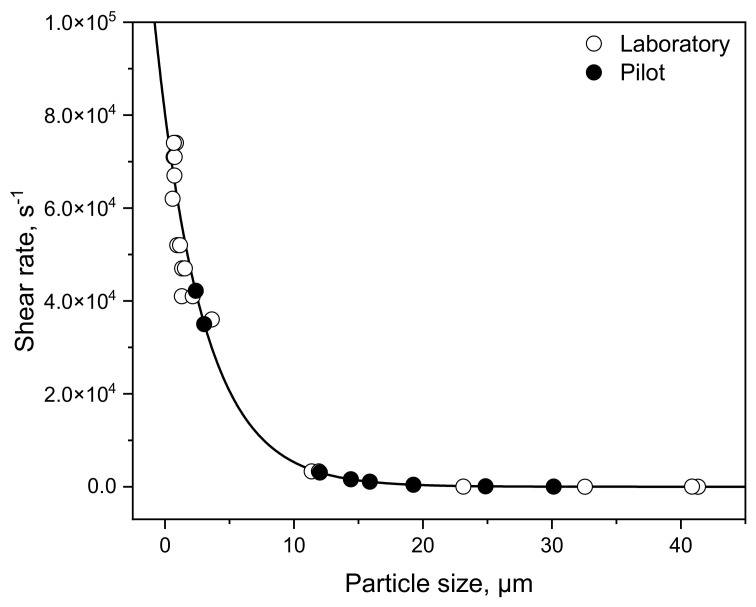
Correlation between calculated shear rates and observed particle size in laboratory scale of MC88 and MPC85 (white circles) homogenized via colloid mill. Black circles represent the particle sizes observed in pilot scale after homogenization with shear pump. Corresponding shear rates are calculated with the correlation function. The correlation coefficient *R*^2^ of the fit was ≥0.99.

In both cases, the gel strength decreased further with increasing particle sizes. Due to the clear particle size-dependent trends—independent from laboratory or pilot scale—we wanted to calculate the “true” shear rates appearing in the shear pump based on the laboratory results. The “true” shear rates (meaning the shear rates which actually occurred) in the shear pump seem to be lower than in the colloid mill, although the applied shear rates were calculated in the same way. For the calculations, high shear (colloid mill) and low shear (overhead stirrer) was applied on skim enriched with either MC88 or MPC85 to obtain a fitting curve covering a wide shear rate range. Equation (7) presents the fit equation and Table 3 the corresponding fit parameters. The curve is illustrated in Figure 9.

(7) $$y=y_{0}+a\cdot e^{\left(b\cdot x\right)}$$

Based on the particle sizes, the “true” shear rates of the pilot results could be calculated by Equation (7). Doing so, the pilot results (black circles) correlated perfectly with the laboratory fit (Figure 9).

The following Table 4 shows the corresponding calculated shear rates compared to the targeted shear rates calculated by Equations (2) and (3). At 3.6 × 10^4^ s^−1^ the deviation between the shear rates was lowest with 1.6%. In contrast to that, the deviation at 5.2 × 10^4^ s^−1^ was highest with 74.2%. This means that at this shear rate, the true shear rate was 74.2% lower than desired. Consequently, the particle sizes were larger due to the lower shear intensity than in laboratory scale. In other words, to induce particle sizes of ~1 µm the conditions at pilot scale must be adapted such that the resulting shear rate is 74.4% higher than calculated by Equations (2) and (3). Thus, the shear rate should be adjusted to 3.9 × 10^6^ instead of 5.2 × 10^4^ s^−1^.

HPH can be taken over from laboratory to pilot scale unrestrictedly; the same pressure induces the same shear rate and hence, the same particle size distribution.

## 4. Conclusions

This study demonstrates how the shear rate in laboratory and pilot rotor/stator systems like colloid mill or shear pump and high-pressure homogenizers (HPH) affect the particle sizes and rennet gelation properties of MC88- and MPC85-enriched skim milk.

Applying HPH (100 bar) on poorly soluble dairy powders like MC88 and MPC85 containing high ratios of slow dissolving components appears to be indispensable and sufficient for full powder dissolution. The flow conditions in the rotor/stator systems were insufficient—even at shear rates up to 9.9 × 10^7^ s^−1^—for complete powder destruction; additional cavitation, which occurs in HPH, was required.

Based on the experimental data, we confirmed our hypothesis that remaining powder particles impair the rennet gelation behavior due to the inaccessible proteins in the powder aggregates as well as the gel properties, which could be disturbed by large powder particles. We could show that insufficient powder rehydration prolongs the rennet gelation time. This could result in too early curd cutting in the cheese making process leading to fluctuations in the resulting cheese properties and quality. Although the gelation time of MC88- and MPC85-enriched skim milk was similar, the addition of MPC85 resulted in softer, weaker gels and consequently, in a reduced syneresis/serum loss. This could be favorable for producing cheeses with high moisture contents. Moreover, remaining powder particles decrease the whey protein concentration in the sweet whey. This can reduce the whey protein yield upon sweet whey purification.

Using HPH is an effective option for powder redispersion, already commonly established in dairies for milk homogenization. Depending on powder characteristics, it could also serve as a method to more effectively redisperse and fully rehydrate more challenging milk specialty powders like MCs or MPCs. Possibly, a toothed disc powder mixer could or would have be installed upstream to HPH in such cases.

## Figures and Tables

**Table 1:** Powder compositions of MC88 and MPC85 (mean ± standard deviation).

	Casein (%, *w*/*w*)	Whey Protein (%, *w*/*w*)	Total Protein (%, *w*/*w*)	Casein/Whey Protein Ratio	Degree of Whey Protein Denaturation (%)	Lactose (%, *w*/*w*)	Minerals (%, *w*/*w*)	Total solids (%)
MC88	78.4 ± 0.5	5.6 ± 0.6	84	93:7	65.4 ± 0.8	1.4 ± 0.2	2.7 ± 0.0	94.4 ± 0.0
MPC85	70.5 ± 0.2	11.5 ± 0.1	82	86:14	42.7 ± 0.0	2.7 ± 0.0	2.4 ± 0.0	94.6 ± 0.0

**Table 2:** Configurations chosen for shear treatments in laboratory and pilot scale for the same shear rate calculated with Equations (1)–(4).

	Stirred Tank	Colloid Mill/Shear Pump	Colloid Mill/Shear Pump	Colloid Mill/Shear Pump	Colloid Mill/Shear Pump	High-Pressure Homogenizer
Configuration laboratory scale	53 rpm	3170 min^−1^	3487 min^−1^	4026 min^−1^	5088 min^−1^	100 bar
Configuration pilot scale	33 rpm	1255 min^−1^	1381 min^−1^	1594 min^−1^	2015 min^−1^	100 bar
Calculated shear rate (s^−1^)	27	3.3 × 10^3^	3.6 × 10^4^	4.1 × 10^4^	5.2 × 10^4^	6.0 × 10^7^

**Table 3:** Fit parameters *y*_0_, *a*, and *b* of modeling the shear rate in laboratory scale by the fit equation.

*y*_0_	*a*	*b*
7.32755	8.034 × 10^4^	−0.27206

**Table 4:** Shear rates in the shear pump calculated with fit Equation (7) (mean of MC88 and MPC85 ± standard deviation) compared to targeted shear rates calculated by Equations (2) and (3). The shear rate deviation between calculated and targeted (percentage of the targeted shear rate) specifies by which shear rate the shear rate calculated by Equations (2) and (3) had to be increased to reach the targeted shear rates of the pilot scale conditions. This gives the adjusted shear rate settings.

Shear Rate Calculated by Equation (7) (s^−1^)	Targeted Shear Rate Calculated by Equations (2) and (3) (s^−1^)	Shear Rate Difference (s^−1^)	Shear Rate Deviation (%)	Adjusted Shear Rate Settings (s^−1^)
2.3 × 10^2^ ± 2.0 × 10^2^	3.3 × 10^3^	3.1 × 10^3^ ± 2.0 × 10^2^	7.0 ± 6.1	2.3 × 10^4^ ± 2.9 × 10^4^
5.9 × 10^2^ ± 4.9 × 10^2^	3.6 × 10^4^	3.5 × 10^4^ ± 4.9 × 10^2^	1.6 ± 1.4	5.9 × 10^4^ ± 6.9 × 10^4^
2.3 × 10^3^ ± 7.3 × 10^2^	4.1 × 10^4^	3.9 × 10^4^ ± 7.3 × 10^2^	5.7 ± 1.8	2.3 × 10^5^ ± 1.0 × 10^5^
3.9 × 10^4^ ± 3.6 × 10^3^	5.2 × 10^4^	1.3 × 10^4^ ± 3.6 × 10^3^	74.2 ± 6.9	3.9 × 10^6^ ± 5.1 × 10^5^

## Data Availability

The data presented in this study are available on request from the corresponding author.
